# A novel preoperative image-guided localization for small pulmonary nodule resection using a claw-suture device

**DOI:** 10.1038/s41598-023-46365-9

**Published:** 2023-11-02

**Authors:** Lijie Wang, Jinxian He, Liang Zhang, Chengcheng Chen, Biao Chen, Weiyu Shen

**Affiliations:** 1 Department of Thoracic Surgery, Ningbo Medical Center Lihuili Hospital, Ningbo University, 1111 Jiangnan Road, Ningbo, 315040 Zhejiang China; 2 Department of Respiration, Ningbo Medical Center Lihuili Hospital, Ningbo University, Ningbo, 315040 Zhejiang China; 3 Department of Radiology, Ningbo Medical Center Lihuili Hospital, Ningbo University, Ningbo, 315040 Zhejiang China

**Keywords:** Surgical oncology, Cancer imaging

## Abstract

Video-assisted thoracoscopic surgery (VATS) provides better option concerning pathological diagnosis and curative intention of small pulmonary nodules (SPNs) that are sometimes challenging to localize. We assess the safety and feasibility of a new localization technique for SPNs, and report experience accumulated over time. A retrospective review of the new claw-suture localization cases between February 2018 and May 2023 was performed. Nodules were localized by a novel system that has an anchor claw and a tri-colored suture, guided by computed tomography (CT). Localization and operative procedure outcomes were then assessed. A total of 590 SPNs were localized from 568 patients before operation. The median nodule size was 0.70 cm (range, 0.3–2.0 cm). The claw-suture localization was successful without dislodgment or device fracture in 574 of 590 lesions (97.3%). Failures included not meeting target distance between claw and lesion (n = 13 [2.2%]), and device displacement (n = 3 [0.5%]). Complications requiring no further medical intervention included asymptomatic pneumothorax (n = 68 [11.5%]), parenchymal hemorrhage (n = 51 [8.6%]), and hemothorax (n = 1 [0.2%]) with the exception of pleural reaction observed in 2 cases (0.3%). Additionally, the depth of pulmonary nodules was significantly associated with the occurrence of pneumothorax (*P* = 0.036) and parenchymal hemorrhage (*P* = 0.000). The median duration of the localization was 12 min (range, 7–25 min). No patient complained of remarkable pain during the entire procedure. Retrieve of device after operation was 100%. The new localization technique is a safe, feasible, and well-tolerated method to localize SPNs for VATS resection.

## Introduction

With low-dose, high-resolution computed tomography (CT) being used for screening of lung cancer widely, small pulmonary nodules (SPNs) are detected especially in high-risk populations^1^. More importantly, up to 50% of these nodules are suspected to be malignant^2^. Therefore, a precise diagnosis is usually needed for these nodules, particularly with ground-glass appearance on CT image.

Traditional approaches for diagnosing SPNs by needle biopsy are reported with a wide range of limitations and diagnosis rates^3,4^. Percutaneous needle biopsy is frequently associated with a risk of pneumothorax, and tends to fail due to inadequate or missed sampling of nodule tissue^5^. Video-assisted thoracoscopic surgery (VATS) may provide better option concerning pathological diagnosis and curative intention^6^. However, identification of target SPNs during VATS is still challenging. The failure of visualizing or palpating a pulmonary nodule has been reported to occur in 54% to 63% of patients without preoperative localization, thus may lead to conversion from VATS to thoracotomy^7^. The rate of conversion has been increased to 63% when the nodules are less than 1 cm in size or deeper than 0.5 cm from the pleural surface or with a current ground-grass opacity (GGO) or semisolid appearance^8^. Several techniques have been put forward to solve this problem, such as preoperative CT-guided localization (hook-wire, microcoils), percutaneous injection of liquid agents (methylene blue, lipiodol), intra-operative ultrasound (US) and electromagnetic navigation bronchoscopy (ENB)^7,9–11^. Among these techniques, the hookwire is most commonly used for the preoperative localization of SPNs owing to its relatively high successful rate of localization (80.6–99.6%)^12^. However, it is often associated with a risk of complications, including pneumothorax, parenchyma hemorrhage, hemothorax, dislodgement, chest pain, vasovagal syncope, and even systemic air embolism^13,14^. Microcoil is also utilized for preoperative localization, and is beneficial as patients experience less discomfort and lower complications compared to hookwires. However, fluoroscopy is usually demanded intraoperatively, which exposes both the patient and surgeon to radiation^15^.

We report our experience using a new device of a claw-suture system to localize SPNs. We report our technique process, localization success rate, complications, and experience accumulated over time. This technique provides a safe and feasible method for localizing SPNs.

## Methods

This retrospective study was performed in accordance with the principles outlined in the Declaration of Helsinki and approved by the Ethical Committee and the Institutional Review Board of Ningbo Medical Center Lihuili Hospital (No. QT2023PJ036). Informed consent was obtained from all patients before preoperative localization as well as the utilization of their personal data.

### Patients

From February 2018 to May 2023, a total of 568 consecutive patients who underwent preoperative localization with the new claw-suture device in our hospital were retrospectively reviewed. Patients were deemed candidates for preoperative localization as follows: (1) Patients had one or more pulmonary nodules with suspected malignancy and had a desire for localization and VATS resection; (2) the nodules were deeper than 1 cm from the pleural surface within the lung tissue; (3) the size of the nodules were less than 2 cm and deemed to video-assisted thoracoscopic surgery resection; (4) the CT image showed ground-glass opacity or semisolid appearance. (5) imaging examinations showed no evidence of advanced malignancy. The exclusion criteria included patients with severe comorbidities, severe coagulation disorders, the presence of severe pulmonary hypertension, advanced disease or distant metastasis. 8 patients were actually excluded. Five of them could not lie flat more than 5 min, the other three were allergic to local anesthetic.

### CT-guided localization

**Figure 1:**
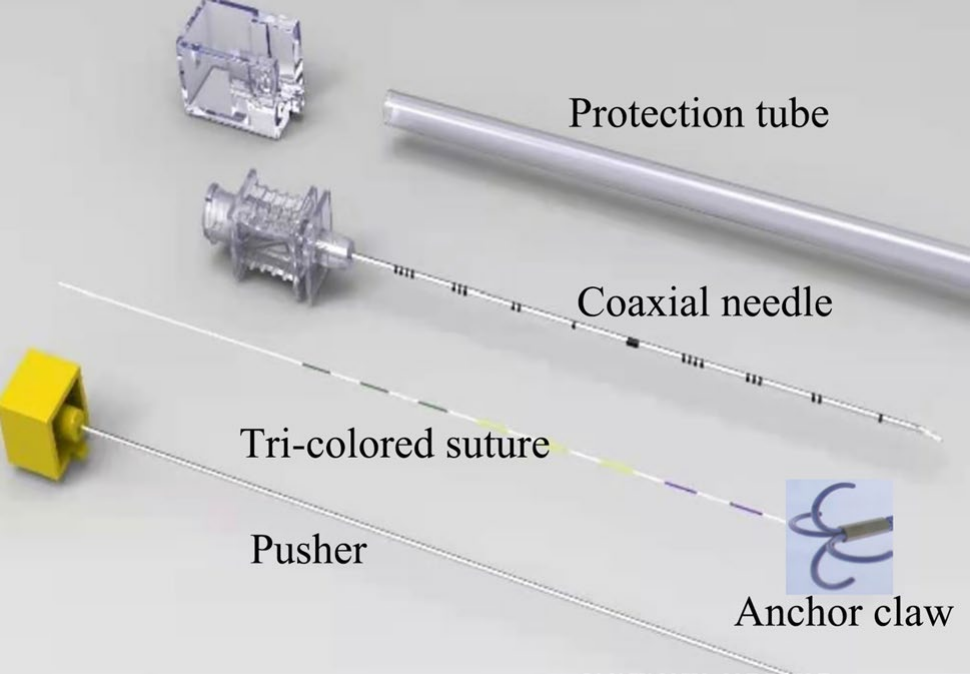
The device includes anchor claw, tri-colored suture, coaxial needle, pusher, and protection tube.

Before VATS resection, all of cases were reviewed by thoracic surgeons and experienced interventional radiologists to determine candidacy of patient. All of localizations were performed by two radiologists who had more than 5 years of intervention experience. After localization, patients underwent resection of SPNs. Patients who did not complete the localization and VATS resection were excluded. This new claw-suture device was designed and manufactured by department of Thoracic Surgery, Shanghai Chest Hospital, and a biotechnology company (Senscure, Ningbo, China), and the details have been introduced in previous study^16^. The device includes anchor claw, tri-colored suture, coaxial needle, pusher, and protection tube (Fig. 1). The main characteristics of this localization device are an 4-hook anchor claw and a tri-colored suture.

**Figure 2:**
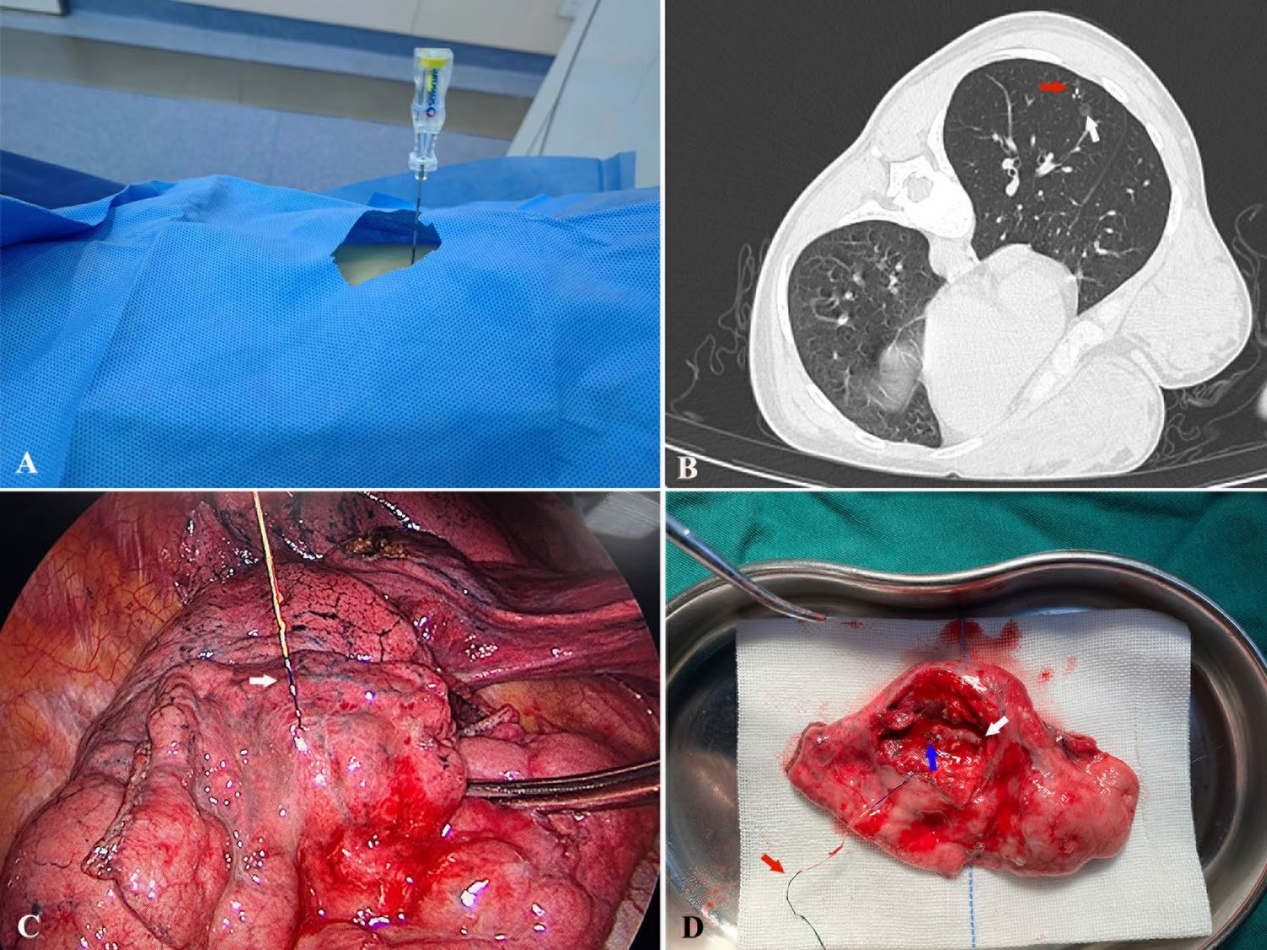
The main process of localization for patients with SPNs. (**A**) The coaxial needle was inserted through the chest wall based on the location of the pulmonary nodule after CT scan. (**B**) CT image shows the pulmonary nodule (white arrow) and the claw (red arrow). (**C**) The tri-colored suture (white arrow) was displayed during the first exploration by VATS. (**D**) The claw (blue arrow) and the tri-colored suture (red arrow), and the localized pulmonary nodule (white arrow) were displayed after wedge resection.

The process of localization was as follows (Fig. 2): (1) The patient was positioned properly based on the location of the pulmonary nodule, and a CT scan was then performed after placement of a metal mesh to determine the skin puncture site; (2) the best puncture site and route were then determined by location of the nodule; (3) 5% lidocaine was injected at the puncture site after sterile preparation of the patient. the coaxial needle was gradually inserted through the wall of the chest; (4) a CT scan was performed again to determine that the needle was in the right position, and then the tip of the needle was adjusted and inserted to the expected position; (5) once the needle was positioned, the claw-suture device was released, the coaxial needle was withdrew, and the final CT scan was then performed to confirm the exact position of the claw-suture device and observe immediately whether there were any complications. VATS resection was performed within 24 h after localization.

### Surgical procedure

All of the enrolled patients were under general intravenous anesthesia, placed in appropriate position, and ventilated using a single or double-lumen endotracheal tube for single lung ventilation. During the first VATS attempt, sutures were pulled into the pleural cavity. Gentle operation of sutures in the pleural cavity allowed for nodule traction in a direction which was be beneficial to VATS resection. The nodules, containing the entire length of the claw-suture device were removed by wedge resection when located in a superficial position. If necessary, segmentectomy was needed due to deep nodules, or unguaranteed adequate negative margins. Segmentectomy was also selected for patients with inadequate pulmonary function to tolerate a lobectomy. All of the nodules were then sent for intraoperative frozen pathology. If invasive lesions or a solid: GGO ratio > 50% were confirmed by frozen pathology, a lobectomy and systemic (or selective) lymph node dissection were conducted.

### Evaluation

The patient clinical data, such as sex, age, pathology, characteristics of nodules, and localization process were collected. The localization success rate, the complication, localization time, and discomfort (chest pain) were assessed through the entire process until the completion of VATS. The localization success should meet following criteria: (1) The distance between the nodule and claw was no more than 1 cm, which was measured as the shortest linear distance from the edge of the nodule to the claw; (2) during the entire process, the device should be placed and retrieved smoothly, and no claw or suture fracture occurred; (3) there was no dislodgement or displacement from the process until nodule resection was completed. Dislodgement was defined as movement of the claw out of the lung tissue into the pleural space. Movement of the claw from the original site was defined as displacement. The pain visual analog scale was used to quantify the pain intensity after localization.

### Statistical analyses

The quantitative data were described as numbers, median, and range. A categorical variables were expressed as numbers and percentages. Bivariate correlation analyses were performed using Pearson correlation analysis. The statistical analyses were performed with SPSS Statistics 23.0 (IBM Corporation, Armonk, NY). A *p* value less than 0.05 was considered to be statistically signifificant for all the analyses.

## Results

### Patient and lesion characteristics

A total of 568 patients (177 male and 391 female; median age, 53 years; age range, 15–82 years) with 590 SPNs who underwent the localization before operation were retrospectively reviewed in the study. Clinical characteristics are shown in Table 1.

**Table 1:** Clinical characteristics of patients with small pulmonary nodules for localization (N = 568).

Variables	n (%)
Total patients	568
Sex	
Male	177 (31.2)
Female	391 (68.8)
Age, y, median (range)	53 (15–82)
Smoking history	
Yes	155 (27.3)
No	413 (72.7)
Time between localization and surgery, h, median (range)	
Immediately* (n = 232)	1.0
Not immediately (n = 336)	20 (2–24)
Patient’s comfort (pain)	
Normal	565
Mild pain	3
Moderate to severe pain	0

Data are expressed as n (%) unless otherwise indicated.

*The time interval immediately after localization was assessed as 1.0 h.

The nodules were classified as solid (50 [8.5%]), GGO (474 [80.3%]), and semisolid (66 [11.2%]). The median nodule size was 0.70 cm (range, 0.3–2.0 cm). The median depth of nodule from visceral pleura was 0.90 cm (range, 0–6.0 cm). All of nodules were resected at the first attempt, which was then confirmed by intraoperative frozen pathology. 518 nodules (87.8%) were removed by wedge resection, 67 lesions (11.4%) by segmentectomy. The remaining five procedures (0.8%) were subsequently converted to lobectomies due to the invasive lesions and a solid: GGO ratio > 50% confirmed by frozen pathology. Pathological diagnosis included primary lung malignancy (494 [83.7%]), metastasis (1 [0.2%]), and benign lesions (95 [16.1%]). All of patients underwent VATS resection with negative margins (Table 2).

**Table 2:** Clinical and pathologic characteristics of small pulmonary nodules for localization (N = 590).

Variables	n (%)
Total nodules	590
Size, diameter, cm, median (range)	0.7 (0.3–2.0)
0–0.5	103 (17.5)
0.6–1.0	412 (69.8)
1.1–1.5	67 (11.3)
1.6–2.0	8 (1.4)
Nodule characteristics	
GGO	474 (80.3)
Solid	50 (8.5)
Semisolid	66 (11.2)
Location	
Right upper lobe	188 (31.9)
Right middle lobe	40 (6.8)
Right lower lobe	129 (21.9)
Left upper lobe	144 (24.4)
Left lower lobe	89 (15.1)
Depth of nodule from visceral pleura, cm, median (range)	0.9 (0–6.0)
Resection	
Wedge resection	518 (87.8)
Segmentectomy	67 (11.4)
Lobectomy	5 (0.8)
Pathological diagnosis	
Adenocarcinoma of the lung	
Adenocarcinoma in situ	93 (15.8)
Minimally invasive adenocarcinoma	344 (58.3)
Invasive adenocarcinoma	48 (8.1)
Mucinous adenocarcinoma	5 (0.8)
Squamous cell carcinoma	2 (0.3)
Primary lung carcinoid	1 (0.2)
Lymphoepithelioma-like carcinoma	1 (0.2)
Metastatic lesions	1 (0.2)
Benign lesions	
Fibrosis scar tissue	46 (7.8)
Granuloma	24 (4.1)
Hamartoma	4 (0.7)
Lymphoid hyperplasia	11 (1.9)
Atypical adenomatous hyperplasia	9 (1.5)
Sclerosing hemangioma	1 (0.2)

Data are expressed as n (%) unless otherwise indicated.

* GGO* ground-glass opacity.

### Localization and surgical procedure outcomes

**Figure 3:**
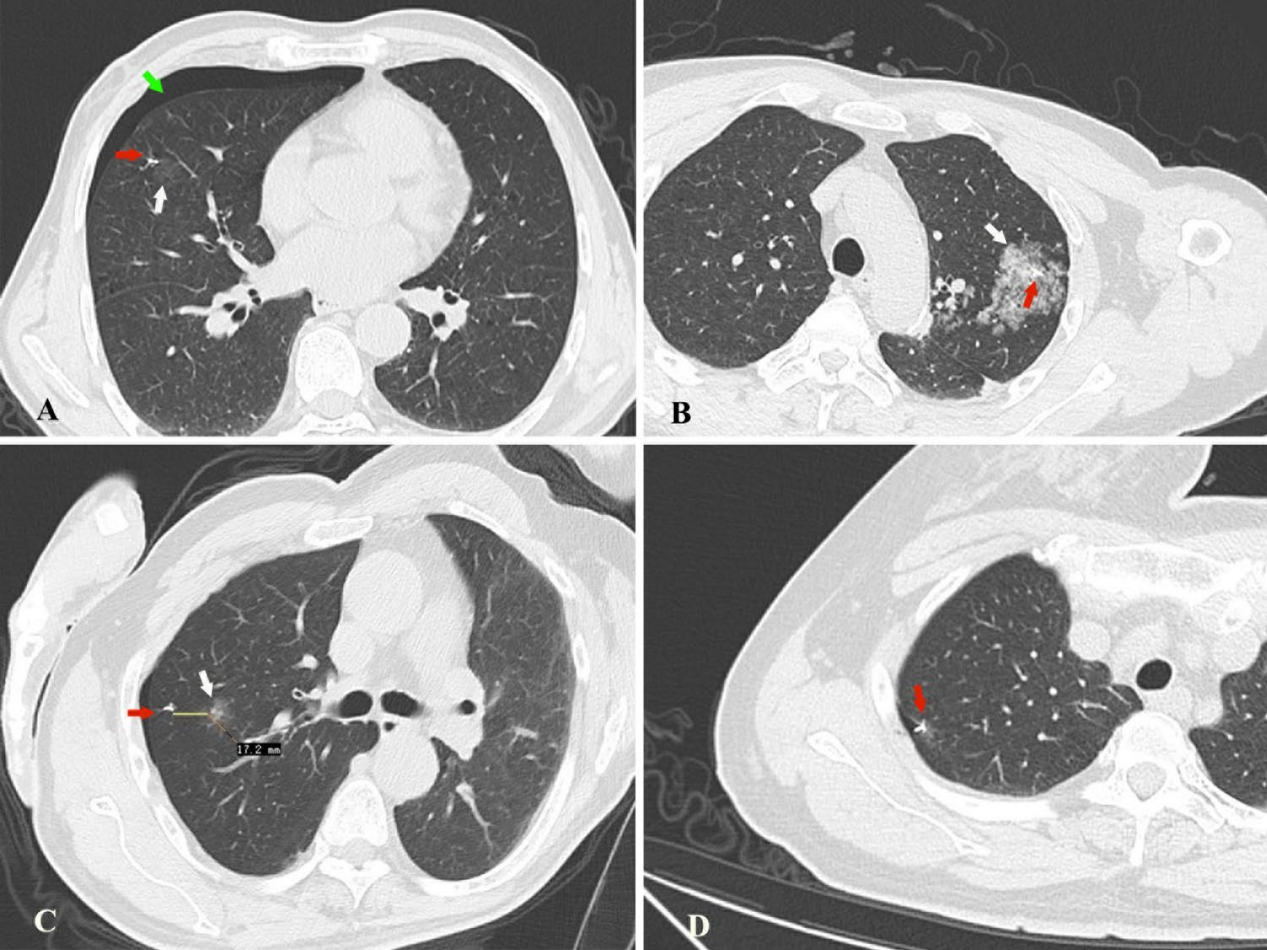
Complications and unsuccessful localization. (**A**) CT image shows asymptomatic pneumothorax (green arrow) with the claw (red arrow) and nodule (white arrow) in right middle lobe. (**B**) CT image shows asymptomatic parenchymal hemorrhage (white arrow) with the claw (red arrow) in left upper lobe. (**C**) CT image shows the distance between the nodule and claw was more than 1 cm with the claw (red arrow) and nodule (white arrow) in right upper lobe. (**D**) CT image shows displaced claw (red arrow) pushing against the lung tissue in right upper lobe.

There were no major complications or deaths occurring during the entire process. Complications associated with the new localization included asymptomatic pneumothorax (68 [11.5%]), parenchymal hemorrhage (51 [8.6%]), and hemothorax (1 [0.2%]) (Fig. 3). Notably, pleural reaction requiring further medical intervention was observed in 2 cases (0.3%). The claw was placed at a median distance of 0.2 cm (range, 0–3 cm) from the edge of the nodule (Table 3). In additionally, the depth of pulmonary nodules was significantly associated with the occurrence of pneumothorax (*P* = 0.036) and parenchymal hemorrhage (*P* = 0.000) (Table 4). Three patients complained of mild chest pain while waiting for the operation without any intervention (Table 1).

**Table 3:** Characteristics of localization procedure (N = 590).

Variables	n (%)
Time of localization procedure, min, median (range)	12 (7–25)
Location of the anchor claw	
Inside of nodules	238 (40.3)
Around nodules	352 (59.7)
Distance between claw and lesion, cm, median (range)	0.2 (0–3.0)
Length of claw-suture in the parenchyma, cm, median (range)	1.2 (0–6.0)
Depth of nodule from visceral pleura	
0–0.5	94 (15.9)
0.6–1.0	171 (29.0)
1.1–1.5	151 (25.6)
1.6–2.0	86 (14.6)
2.1–3.0	73 (12.4)
3.1–6.0	15 (2.5)
Successful localization	574 (97.3)
Unsuccessful localization	16 (2.7)
Distance between claw and lesion > 1.0 cm	13 (2.2)
Displacement	3 (0.5)
Dislodgement	0 (0)
Device fracture	0 (0)
Complications	
Pneumothorax	
Asymptomatic	68 (11.5)
Symptomatic	0 (0)
Parenchymal hemorrhage	
Asymptomatic	51 (8.6)
Symptomatic	0 (0)
Hemothorax	
Asymptomatic	1 (0.2)
Symptomatic	0 (0)
Pleural reaction	2 (0.3)
Retrieve of device after resection	590 (100)

Data are expressed as n (%) unless otherwise indicated.

**Table 4:** Pearson correlation analysis of localization procedure and complications.

Variables		Pneumothorax	Parenchymal hemorrhage
Depth of nodule from visceral pleura	rs	0.086	0.208
	*P*-value	0.036	0.000
Length of claw-suture in the parenchyma	rs	0.088	0.189
	*P*-value	0.033	0.000

*rs* Pearson correlation coefficient.

The localization was successful in 574 of 590 lesions (97.3%). 21 patients with multiple nodules underwent simultaneous localization. Of these localization procedures, double localization was successful in 20 patients, including 8 performed in the same lobe and 12 performed in 2 different lobes. Placement also succeeded in one patient with 3 lesions located in 2 different lobes. All of devices were visualized at the first attempt before VATS resection and retrieved entirely with the resected nodules (Table 3).

Of the sixteen cases which had unsuccessful localization, 13 cases were further away from the nodules. The distance between the claw and lesion was 1.1–3.0 cm, which was further from the target distance (1.0 cm). Other three unsuccessful cases were due to displacement because the nodules were adjacent to the surface of the visceral pleura that the needle could not puncture through the pleura but still held on the surface of pleura (Fig. 3). All of these nodules were completely resected by VATS. There was no cases of dislodgement or claw-suture fracture during the entire process (Table 3).

In this study, the median duration of the process was 12 min (range, 7–25 min) (Table 3). There were 232 patients (40.8%) who underwent surgery immediately after localization procedure. The time interval was assessed at approximately 1.0 h, and 336 patients (59.2%) had a median time interval between localization and surgical procedure, which was 20 h (range, 2–24 h) (Table 1). The delay was due to a large number of surgery schedules, complicated surgery ahead and complications.

## Discussion

While there is an increasing role for VATS resection of SPNs for both diagnostic and curative purposes, surgeons are still faced with challenges as the nodules are often impalpable and invisible in the first VATS attempt^14^. Our study shows that the novel localization with a claw-suture device is a safe and feasible method of SPNs localization with a high success rate.

Currently, most surgeons have usually relied on three basic methods for SPNs localization: (1) Image-guided percutaneous needle localization; (2) ENB dye marking or needle placement; and (3) intraoperative US technique^17^.

The commonly used percutaneous needle localization is hookwire localization. The most common complication associated with this technique is pneumothorax, the rate of which ranges between 7.5 to 40%^18^. In addition, parenchymal hemorrhage has frequently been reported (16%), while dislodgement occurs in 2.5–13% of cases^19^. However, a rare major complication of systemic air embolism has been reported with a rate of 0.6% in a large case report^20^. Moreover, the technique was originally designed for breast nodule localization, which might not be the optimal choice for pulmonary lesions.

ENB-guided dye marking has gained a lot of popularity in recent years^21^. Localization by ENB-guided dye marking proved to be effective and significantly reduce dye diffusion and the risk of pneumothorax compared with the conventional percutaneous marking process^22^. Song et al. reported successful localization in 94.5% of 164 pulmonary lesions with no ENB-related complications observed^23^. However, the patients still receive radiation from intraoperative CT image, and these ENB systems are not viable for rural or low-income areas, and access to equipment and training is limited in hospitals.

Intraoperative US techniques have been widely used for localizing pulmonary nodules since the 1990s. Many clinicians have reported that US is a safe and effective option allowing intraoperative SPNs identification in up to 93% of cases^24,25^. Nonetheless, nodules with GGO or semisolid appearance were difficult to identify^26^. Moreover, as the US technique demands that the lung should be completely deflated, as localization in patients with emphysema was showed to be more difficult, and US process is highly dependent on operator that only experienced interventional radiologists could localize these nodules effectively and safely^27^.

As far as we know, this is one of the largest series of the new localization performed to date. Our localization experience for SPNs has been a multidisciplinary effort for more than 5 years. We share our cumulative experience that we hope will aid other thoracic surgeons. First, unlike the hookwire which keeps a length of steel wire outside the body after localization, we push the soft suture that is attached to the claw into thorax, which may significantly reduce the tension of the chest wall to the claw caused by position change or respiratory movement, as we have seen more dislodgement with the wires firmly attached to the chest wall. Second, we find that the 4-hook anchor claw is the most important component of this new device. When the claw is gradually released, it can hook the adjacent tissue without causing a great structural damage, remarkably reducing the risk of pneumothorax and parenchymal hemorrhage. Third, as the claw can endure considerable traction during the operation, we can pull the suture up to lift the adjacent lung tissue to create a better visual field exposure, which makes it convenient to resect the lesions and maximize preservation of lung function. Fourth, the scale-coaxial needle assists surgeons regarding precise localization. One of the important processes is to take out the pusher, then we withdraw the scale-coaxial needle until the end is within 0.5–1 cm from the inner surface of the chest wall so that the tip of the needle can be exposed in the pleural cavity. Cases in which the nodules were adjacent to the visceral pleura need to be dealt with carefully. The end of the coaxial needle should be pushed a little further beyond the nodule to avoid failure of breaking through the visceral pleura, or “pseudo-pneumothorax” caused by the needle pushing against the tissue. Fifth, we find tri-color suture is a reliable marker that helps evaluating depth of the nodules, ensures adequate negative margins during wedge resections and segmentectomy. However, we found the depth of pulmonary nodules and the length of the suture in the lung tissue was significantly associated with the occurrence of parenchymal hemorrhage. We suggest that surgeons should always be aware of the risk of parenchymal hemorrhage for nodules deep in the lung tissue. Sixth, we have found that the coaxial needle was inevitably placed at an acute angle to the surface of the visceral pleura while localization was in the apex or diaphragm. In these cases, the claws were more likely to be displaced to the surface of the lung tissue and make VATS resection more difficult. Moreover, if segmentectomy or lobectomy is not performed, the entry point for displaced claw should be included in the resected lesion to avoid continuous air leakage. Finally, we recommend that the placement of the claw should be adjacent to the nodule. Localization deeper than the heart-side of the nodule within the lung tissue often leads to unnecessarily deep wedge resections that may distort the lobe, compromise lung function, and make further anatomic resection complicated.

The limitation of our study is that it is a retrospective, nonrandomized, and single-center analysis of a selected group of patients. Further prospective large-sample randomized clinical trials are warranted to confirm the safety and effectiveness of this technique. We demonstrates this CT-guided localization with claw-suture placement is a feasible and safe technique that allows for precise resection of SPNs by VATS. There are other advantages compared with the current localization techniques, such as hookwire, microcoil, lipiodol, dyes, and radiotracer^7,9,10^. Patients can move freely during the waiting period, and the device can stay in the body relatively longer without causing obvious discomfort^28^. Furthermore, this technique is easy to operate while the device can be available in most hospitals, and has the ability to be potentially applied to almost the whole lung field with no radiation exposure to surgeons.

In conclusion, the novel localization with a claw-suture system is a safe, feasible, and well-tolerated method for localizing SPNs that are difficult to localize before VATS resection.

## Data Availability

The datasets generated and/or analysed during the current study are not publicly available in order to protect the privacy of patients but are available from the corresponding author on reasonable request.
